# Diaphragmatic dysfunction associates with dyspnoea, fatigue, and hiccup in haemodialysis patients: a cross-sectional study

**DOI:** 10.1038/s41598-019-56035-4

**Published:** 2019-12-18

**Authors:** Bin Wang, Qing Yin, Ying-yan Wang, Yan Tu, Yuchen Han, Min Gao, Mingming Pan, Yan Yang, Yufang Xue, Li Zhang, Liuping Zhang, Hong Liu, Rining Tang, Xiaoliang Zhang, Jingjie xiao, Xiaonan H. Wang, Bi-Cheng Liu

**Affiliations:** 1 Institute of Nephrology, Zhong Da Hospital, Southeast University School of Medicine, Nanjing, Jiangsu China; 2 Department of Ultrasound Medicine, Zhong Da Hospital, Southeast University School of Medicine, Nanjing, Jiangsu China; 3 Department of Oncology, Department of Agricultural, Food and Nutritional Science, University of Alberta, Edmonton, Canada; 4 Department of Medicine, Renal Division, Emory University, Atlanta, Georgia United States of America

**Keywords:** Adaptive clinical trial, Haemodialysis

## Abstract

Muscle wasting is associated with increased mortality and morbidity in chronic kidney disease (CKD) patients, especially in the haemodialysis (HD) population. Nevertheless, little is known regarding diaphragm dysfunction in HD patients. We conducted a cross-sectional study at the Institute of Nephrology, Southeast University, involving 103 HD patients and 103 healthy volunteers as normal control. Ultrasonography was used to evaluate diaphragmatic function, including diaphragm thickness and excursion during quiet and deep breathing. HD patients showed lower end-inspiration thickness of the diaphragm at total lung capacity (0.386 ± 0.144 cm vs. 0.439 ± 0.134 cm, p < 0.01) and thickening fraction (TF) (0.838 ± 0.618 vs. 1.127 ± 0.757; p < 0.01) compared to controls. The velocity and excursion of the diaphragm were significantly lower in the HD patients during deep breathing (3.686 ± 1.567 cm/s vs. 4.410 ± 1.720 cm/s, p < 0.01; 5.290 ± 2.048 cm vs. 7.232 ± 2.365 cm; p < 0.05). Changes in diaphragm displacement from quiet breathing to deep breathing (△m) were lower in HD patients than in controls (2.608 ± 1.630 vs. 4.628 ± 2.110 cm; p < 0.01). After multivariate adjustment, diaphragmatic excursion during deep breathing was associated with haemoglobin level (regression coefficient = 0.022; p < 0.01). We also found that the incidence of dyspnoea and hiccup and the fatigue scores, all of which were related to diaphragmatic dysfunction, were significantly higher in HD patients than in controls (all p < 0.01). Improving diaphragm function through targeted therapies may positively impact clinical outcomes in HD patients.

## Introduction

Chronic kidney disease (CKD) not only has a decline in kidney function but also affects other organs, such as the respiratory system^1^. In fact, dialysis patients often experience muscle weakness and atrophy that may be related to anaemia, protein/energy imbalance, malnutrition, decreased serum calcium levels, and reduced resistance to oxidative stress^2,3^. Muscle wasting is associated with increased mortality and morbidity in CKD patients^4^. Whereas limb skeletal muscle has traditionally been the main focus, the characteristics of respiratory muscles and the clinical implications of changes in these muscles under CKD conditions have been less investigated.

The diaphragm is the most important respiratory muscle, accounting for 60–80% of respiration^5^. Diaphragmatic dysfunction is prevalent in many diseases, including chronic obstructive pulmonary disease (COPD), chronic heart failure (CHF) and diseases requiring intensive care, especially mechanical ventilation^6^. Previous studies have shown that patients with CKD have decreased ventilation function^7^. When CKD develops into end-stage renal disease (ESRD), patients must receive haemodialysis (HD), peritoneal dialysis, or kidney transplantation to sustain life. Because 87.7% of ESRD patients choose HD as renal replacement therapy^8^, we primarily focus on diaphragmatic dysfunction in HD patients in the present study. The clinical symptoms of diaphragm dysfunction mainly consist of unexplained dyspnoea (especially in the supine position), fatigue, and hiccups, all of which are prevalent in HD patients^9–11^. Since clinicians usually simplify these nonspecific presentations by ascribing them to assumed impaired heart function or volume overload, diaphragm dysfunction in HD is underdiagnosed. The prevalence of diaphragm dysfunction during HD is unclear, and its significance has not been elucidated.

Several techniques, including fluoroscopy, phrenic nerve stimulation, dynamic magnetic resonance imaging of the diaphragm, and trans-diaphragmatic pressure measurement, can be used to assess diaphragmatic function^12^. However, each of these techniques has its own limitations and drawbacks such as exposure to ionizing radiation, low availability, invasiveness, and the need for patient transportation. Compared to these methods, ultrasound is widely available and has several advantages over other modalities, including the absence of radiation, portability, real-time imaging, non-invasiveness, well-described techniques, and reference values^13^. Diaphragm function, including diaphragm thickness and diaphragm excursion, can be evaluated by instant monitoring using ultrasound^14^.

The primary aim of this study was to quantify diaphragm thickness and excursion in a representative sample of HD patients and to compare it with that of an age- and sex-matched healthy cohort using neuromuscular ultrasound assessment. The secondary objective was to identify the risk factors associated with diaphragm dysfunction and to explore the relationship between some common but nonspecific clinical symptoms (dyspnoea, fatigue, and hiccups) with diaphragm dysfunction in our cohort. In addition, we further confirmed diaphragm dysfunction in an animal model of CKD.

## Results

### Patient characteristics and clinical features

A total of 206 participants were enrolled in this study. Mean age was 53.58 ± 12.96 years; 58.25% of patients were male. As shown in Table 1, Body Mass Index (BMI) was significantly lower in HD patients than in the control group (21.98 ± 3.29 vs. 24.14 ± 3.25; p < 0.01). With respect to factors other than BMI, HD patients showed significantly lower haemoglobin (103.82 ± 20.82 vs. 143.68 ± 16.40; p < 0.01), albumin (37.84 ± 4.47 vs. 45.80 ± 3.15; p < 0.01) and glucose (6.42 ± 2.70 vs. 4.95 ± 0.86; p < 0.01) levels. The incidence of comorbidities and the use of antihypertensive drugs were all significantly higher in the HD patients than in the controls (Table 1).

**Table 1:** Baseline characteristics of all patients (n = 206).

Variable	All subjects (*n* = 206)	Maintenance HD (*n* = 103)	Controls (*n* = 103)	P value
Age (years)	53.58 ± 12.96	54.41 ± 14.09	52.76 ± 11.74	0.362
Sex—no. (%)				1.000
Male	120 (58.25)	60 (58.25)	60 (58.25)	
Female	86 (41.75)	43 (41.75)	43 (41.75)	
Body weight	63.09 ± 12.92	59.93 ± 13.84	66.22 ± 11.14	***0***.***0002***
Height	165.08 ± 8.11	165.31 ± 8.53	164.87 ± 7.71	**0**.**6480**
Body mass index (kg/m^2^)	23.16 ± 3.47	21.98 ± 3.29	24.34 ± 3.25	<***0***.***001***
Smoking—no. (%)	81 (39.32)	41 (39.81)	40 (38.83)	0.887
Alcohol—no. (%)	67 (32.52)	31 (30.10)	36 (34.95)	0.457
**Blood variables**				
Hb (g/L)	124.24 ± 26.44	103.82 ± 20.82	143.68 ± 16.40	<***0***.***001***
Albumin (g/L)	41.96 ± 5.53	37.84 ± 4.47	45.80 ± 3.15	<***0***.***001***
Triglycerides (mmol/L)	1.82 ± 1.89	1.65 ± 1.05	1.97 ± 2.40	0.216
Glucose (mmol/L)	5.66 ± 2.11	6.42 ± 2.70	4.95 ± 0.86	<***0***.***001***
**Comorbidities**				
Hypertension—no. (%)	120 (58.25)	97 (94.17)	23 (22.33)	<***0***.***001***
Coronary heart disease	13 (6.31)	13 (12.62)	0 (0)	<***0***.***001***
Chronic heart failure	18 (8.74)	18 (17.48)	0 (0)	<***0***.***001***
Diabetes mellitus	27 (13.11)	26 (25.24)	1 (0.97)	<***0***.***001***
Hyperparathyroidism	7 (3.40)	7 (6.80)	0 (0)	***0***.***014***
**Drugs**				
CCB	73 (35.44)	61 (59.22)	12 (11.65)	<***0***.***001***
ACEI	27 (26.21)	18 (17.48)	9 (8.74)	0.063
ARB	27 (26.21)	23 (22.33)	4 (3.88)	<***0***.***001***
β blockers	58 (28.16)	48 (46.60)	10 (9.71)	<***0***.***001***
Statins	8 (3.88)	6 (5.83)	2 (1.94)	0.140
Glucocorticoids	2 (0.97)	2 (1.94)	0 (0)	0.498

Hb, Haemoglobin; CCB, Calcium channel blockers; ACEI, Angiotensin-converting enzyme inhibitor; ARB, Angiotensin II receptor antagonist; β blocker, Beta blockers; BMI, Body Mass Index; Comparison of Coronary heart disease, Chronic heart failure, Diabetes mellitus, Hyperparathyroidism, and Glucocorticoids between the two groups was performed using Fisher’s exact test; Comparison of ACEI, ARB and Statins between the two groups was performed using a calibration chi-square test; Comparison of other indicators was performed using the t-test.

### Evidence of diaphragm dysfunction in HD

As shown in Table 2, the thickness of the diaphragm at functional residual capacity (FRC) and residual volume (RV) was similar in the two groups. However, the thickness of the diaphragm at total lung capacity (TLC) was lower in HD patients than in controls (0.39 ± 0.14 cm vs. 0.44 ± 0.13 cm, p < 0.01); HD patients also showed a lower TF (0.84 ± 0.62 vs. 1.13 ± 0.76; p < 0.01). Although the velocity and extent of diaphragm excursion was comparable during quiet breathing, it was significantly lower in the HD patients during deep breathing (3.69 ± 1.57 cm/s vs. 4.41 ± 1.72 cm/s, p < 0.01; 5.29 ± 2.05 cm vs. 7.23 ± 2.37 cm; p < 0.05). △m was lower in HD than in controls (2.61 ± 1.63 cm vs. 4.63 ± 2.11 cm; p < 0.01).

**Table 2:** Comparison of diaphragmatic values in HD patients and controls.

Variables	All patients (*n* = 206)	Maintenance HD (*n* = 103)	Controls (*n* = 103)	P value
**Diaphragm thickness**				
TdiFRC (cm)	0.219 ± 0.073	0.218 ± 0.073	0.219 ± 0.073	0.929
TdiVT (cm)	0.283 ± 0.097	0.283 ± 0.095	0.283 ± 0.099	0.989
TdiRV (cm)	0.205 ± 0.068	0.211 ± 0.077	0.198 ± 0.056	0.167
TdiTLC (cm)	0.413 ± 0.142	0.386 ± 0.144	0.439 ± 0.134	***0***.***007***
TF	0.983 ± 0.704	0.838 ± 0.618	1.127 ± 0.757	***0***.***003***
**Excursion and velocity of diaphragm**				
DMFRC (cm)	2.643 ± 1.014	2.682 ± 1.007	2.604 ± 1.026	0.582
Time1 (s)	1.088 ± 0.300	1.096 ± 0.362	1.079 ± 0.222	0.692
Velocity1 (cm/s)	2.497 ± 0.943	2.543 ± 0.906	2.451 ± 0.981	0.485
DMTLC (cm)	6.261 ± 2.412	5.290 ± 2.048	7.232 ± 2.365	<***0***.***001***
Time2 (s)	1.639 ± 0.563	1.532 ± 0.528	1.746 ± 0.579	***0***.***006***
Velocity2 (cm/s)	4.048 ± 1.681	3.686 ± 1.567	4.410 ± 1.720	***0***.***002***
△m (cm)	3.617 ± 2.136	2.608 ± 1.630	4.628 ± 2.110	<***0***.***001***

Thickening fraction (TF) = ((Thickness at FRC − Thickness at RV)/Thickness at RV) × 100; TdiFRC, End-expiration thickness of the diaphragm at functional residual capacity (FRC); TdiVT, End-inspiration thickness of the diaphragm at tidal volume (VT); TdiRV, End-expiration thickness of the diaphragm at residual capacity (RV); TdiTLC, End-inspiration thickness of the diaphragm at total lung capacity (TLC); DMFRC, Diaphragm excursion at FRC; DMTLC, Diaphragm excursion at TLC;Time1, Time of diaphragm excursion at FRC; Time2, Time of diaphragm excursion at TLC; Velocity1 = DMFRC/Time1; Velocity2 = DMTLC/Time2; △m = DMFRC − DMTLC.

### Factors associated with diaphragm dysfunction

We used a multivariable linear regression model to identify risk factors associated with diaphragm dysfunction in the entire cohort. After multivariate adjustment for factors such as age, sex, height, weight, smoking, Hb, alb, TG, Coronary heart disease (CHD), CHF and glucocorticoid levels (the assignment table is shown in Supplemental Table 1), hemoglobin level may have effects on diaphragmatic functions such as diaphragm excursion at TLC (DMTLC) and △m (regression coefficient = 0.0221316, p = 0.019 and regression coefficient = 0.0223932, p = 0.005, respectively; Table 3). Additionally, TF was associated with the occurrence of CHD (regression coefficient = −0.6434051; p = 0.012; Table 3). Diaphragm thickness during deep breathing was associated with weight (regression coefficient = 0.0032217; p = 0.001; Table 3) and was greater in men than in women. We found that the velocity of diaphragm movement during deep breathing (abbreviated as velocity2) in HD was significantly lower than that of controls. History of smoking showed an associationwith velocity2 (regression coefficient = −0.736901; p = 0.041; Table 3). A few covariates changed P values and it is possibly due to the small sample size in the hemodialysis group (from significant to not significant, Supplemental Table 2); however, the trend is similar as in the entire cohort — hemoglobin showed a trend in its association with diaphragmatic function. No association was found between diaphragm parameters and hs-CRP, drugs or lipid profile.

**Table 3:** Multiple linear regression of diaphragm parameters and other factors in the entire cohort.

	DMTLC		TF		TdiTLC		△m		velocity2	
	Coefficient	*P*	Coefficient	*P*	Coefficient	*P*	Coefficient	*P*	Coefficient	*P*
Age (years)	−0.0167243	0.246	0.0063271	0.166	0.0006493	0.447	−0.0330097	0.008	0.0050538	0.651
Sex	0.3179973	0.593	0.3412101	0.071	−0.07074	***0***.***046***	1.037685	***0***.***041***	0.2747333	0.552
Height (cm)	0.0254866	0.460	0.0157091	0.151	−0.0026693	0.193	0.0511959	0.082	−0.0114361	0.669
Weight (kg)	0.0177167	0.288	−0.000926	0.861	0.0032217	***0***.***001***	0.0052439	0.711	0.0099112	0.444
Smoking	−0.7391283	0.110	−0.1606524	0.272	−0.0283733	0.300	−0.7293134	0.064	−0.736901	***0***.***041***
Hb (g/L)	0.0221316	***0***.***019***	−9.40e-06	0.997	0.0001671	0.764	0.0223932	***0***.***005***	0.0038262	0.600
Alb (g/L)	0.0400414	0.371	0.0076297	0.590	−0.0019837	0.455	0.0507548	0.182	0.0165361	0.634
TG (mmol/L)	0.015103	0.868	−0.026671	0.355	0.0055287	0.306	0.0331432	0.668	0.0709124	0.316
CHD	−0.2641019	0.741	−0.6434051	***0***.***012***	−0.0501496	0.290	−0.7666822	0.259	−0.1592421	0.797
CHF	−0.7776509	0.274	−0.0856891	0.703	−0.0486362	0.249	−0.1417835	0.814	−0.3716404	0.501
Glucocorticoids	1.559383	0.342	−0.2330599	0.653	−0.1009406	0.300	2.528522	0.071	0.9312895	0.465

Hb, Haemoglobin; BMI, Body Mass Index; Alb, Albumin; TG, Triglycerides; CHD, Coronary heart disease; CHF, Chronic heart failure. All the regression models are adjusted by confounders, and covariates are all presented in the table.

### Diaphragm dysfunction associated with clinical presentations

#### Dyspnoea or shortness of breath

The incidence of dyspnoea was significantly higher in dialysis patients than in controls (27/103 vs. 0/103; p < 0.01; Supplemental Table 3) and was negatively correlated with △m (OR = 0.423938; p < 0.05; Table 4). In a fully adjusted model (the logistic regression assignment table is shown in Supplemental Table 4), the occurrence of CHF was associated with dyspnoea (OR = 29.99488; p < 0.01; Table 4), and serum albumin level was inversely related to dyspnoea (OR = 0.795313; p < 0.05; Table 4). To exclude the influences of dialysis capacity load and heart function, we performed a subgroup analysis in the HD group and found that after adding interdialytic weight gain rate to the model, the results remained the same (Supplemental Table 5).

**Table 4:** A binary logistic regression model of dyspnoea and other factors in the entire cohort.

dyspnoea	OR	[95% Conf.	Interval]	P > |z|
Age (years)	1.007497	0.954692	1.063224	0.786
TdiTLC (cm)	29.52243	0.322847	2699.648	0.142
DMTLC (cm)	1.609243	0.917705	2.821891	0.097
△m (cm)	0.423938	0.194352	0.924732	***0***.***031***
TF	1.399315	0.46116	4.245992	0.553
Hypertension	8.045581	0.492681	131.3859	0.143
Hb (g/L)	1.014508	0.982101	1.047986	0.385
Alb (g/L)	0.795313	0.664316	0.952142	***0***.***013***
Glu (mmol/L)	0.947009	0.733308	1.222985	0.676
CCB	0.993782	0.246977	3.998767	0.993
CHD	2.746759	0.339466	22.22515	0.344
CHF	29.99488	4.149523	216.8183	***0***.***001***
DM	0.382417	0.063395	2.30684	0.294

TdiTLC, End-inspiration thickness of the diaphragm at total lung capacity (TLC); DMTLC, Diaphragm excursion at TLC; DMFRC, Diaphragm excursion at FRC; △m = DMFRC-DMTLC; TF, Thickening fraction; Hb, Haemoglobin; Alb, Albumin; Glu, Glucose; CCB, Calcium channel blockers; CHD, Coronary heart disease; CHF,Chronic heart failure; DM, Diabetes mellitus. All the regression models are adjusted by confounders, and covariates are all presented in the table.

#### Fatigue

Comparison between the two groups showed that the HD patients had higher fatigue scale scores, and the difference was statistically significant (58.65 ± 14.64 vs. 32.14 ± 8.17; p < 0.01; Table 5). Multiple linear regression model analysis found that △m (regression coefficient = −1.534015; p < 0.05; Table 5) was associated with fatigue, suggesting that individuals with diaphragmatic dysfunction had stronger feelings of fatigue. In addition, fatigue scores were associated with albumin and glucose (Table 5). We performed the same analysis in the hemodialysis subgroup, and obtained similar results (Supplemental Table 6).

**Table 5:** Multiple linear regression of fatigue and other factors in the entire cohort.

Fatigue	Coefficient	[95% Conf.	Interval]	P > t
△m (cm)	−1.534015	−2.496955	−0.571075	***0***.***002***
TF	−0.572743	−3.473556	2.32807	0.697
TdiTLC (cm)	−4.598935	−19.80479	10.60692	0.551
Hypertension	3.730772	−1.871455	9.332999	0.191
BMI (kg/m2)	−0.2166063	−0.7991327	0.3659201	0.464
Hb (g/L)	−0.0476258	−0.1470496	0.051798	0.346
Alb (g/L)	−1.230764	−1.693329	−0.7681995	<***0.001***
Glu (mmol/L)	1.225458	0.3060835	2.144832	***0***.***009***
CCB	3.071568	−1.969332	8.112467	0.231
ARB	1.164637	−4.400537	6.729811	0.680
β blockers	−0.550096	−5.443589	4.343397	0.825

DMTLC, Diaphragm excursion at TLC; DMFRC, Diaphragm excursion at FRC; △m = DMFRC-DMTLC; TdiTLC, End-inspiration thickness of the diaphragm at total lung capacity (TLC); TF, Thickening fraction; BMI, Body Mass Index;Hb, Haemoglobin; Alb, Albumin; Glu, Glucose; CCB, Calcium channel blockers; ARB, Angiotensin II receptor antagonist; β blocker, Beta blockers; All the regression models are adjusted by confounders, and covariates are all presented in the table.

#### Hiccups

The incidence of hiccup was significantly higher in HD patients than in controls (15.53% vs. 0.97%; p < 0.01; Supplemental Table 3). Our data analysis showed that the symptoms of hiccup were related to △m. The greater the value of △m, the lower the incidence of hiccup in patients (OR = 0.323599; p < 0.05; Table 6). The same analysis in the hemodialysis subgroup obtained similar results (Supplemental Table 7).

**Table 6:** A binary logistic regression model of hiccups and other factors in the entire cohort.

Hiccups	OR	[95% Conf.	Interval]	P > |z|
DMTLC (cm)	1.471358	0.770888	2.808311	0.242
△m (cm)	0.323599	0.129852	0.806428	***0***.***015***
TdiRV (cm)	21.11987	0.000133	3347320	0.618
TdiTLC (cm)	2.701769	0.01793	407.1223	0.698
Hypertension	0.720038	0.044961	11.53109	0.816
Hb (g/L)	0.977729	0.941824	1.015003	0.238
Alb (g/L)	0.964546	0.809048	1.14993	0.687
Glu (mmol/L)	1.020523	0.809777	1.286117	0.863
CCB	1.828497	0.341026	9.803958	0.481
ARB	2.570176	0.552937	11.94676	0.229
β blockers	0.708931	0.153092	3.282871	0.66
CHD	2.453036	0.247508	24.31192	0.443
CHF	0.916544	0.096162	8.735819	0.94
DM	1.24614	0.266187	5.833739	0.78

DMTLC, Diaphragm excursion at TLC; DMFRC, Diaphragm excursion at FRC; △m = DMFRC-DMTLC; TdiRV, End-expiration thickness of the diaphragm at residual capacity (RV); TdiTLC, End-inspiration thickness of the diaphragm at total lung capacity (TLC); Hb, Haemoglobin; Alb, Albumin; Glu, Glucose; CCB, Calcium channel blockers; ARB, Angiotensin II receptor antagonist; β blocker, Beta blockers; CHD, Coronary heart disease; CHF, Chronic heart failure; DM, Diabetes mellitus. All the regression models are adjusted by confounders, and covariates are all presented in the table.

## Discussion

Our study revealed that HD patients presented decreased diaphragmatic thickness and excursion during deep breathing and lower TF in comparison with control subjects, indicating the presence of diaphragmatic dysfunction in HD patients. To our knowledge, this is the first study in which ultrasound has been used to evaluate diaphragmatic dysfunction in haemodialysis patients. Importantly, we found that diaphragmatic dysfunction was highly prevalent and that it showed a positive association with deleterious consequences and discomforts such as dyspnoea, fatigue and hiccups in HD patients.

In previous studies, we and other nephrologists mainly focused on limb skeletal muscle wasting in CKD^15^. The diaphragm is the main respiratory muscle and plays a key role in respiratory movement. Diaphragm dysfunction is associated with increased mortality and morbidity in a variety of diseases such as COPD, CHF, and diseases requiring intensive care, especially those requiring mechanical ventilation. Since the diaphragm is the largest skeletal muscle in the visceral system and previous studies have shown that CKD results in significantly reduced muscle mass and strength^16^, it is reasonable to hypothesize that muscle wasting also occurs in the diaphragm under CKD stress conditions. Diaphragmatic atrophy is associated with decreased diaphragmatic function^17^; the reduction in muscle fibre length impairs the ability of the diaphragm to produce force and reduces the rate of shortening^18^.

Diaphragmatic dysfunction is suggested by lower-than-normal amplitude of excursion on deep breathing with or without paradoxical motion on sniffing^19^. Diaphragmatic dysfunction can be unilateral or bilateral; unilateral lesions are usually asymptomatic and are often accidentally discovered during the examination^19^. Ultrasound is a non-invasive, feasible and accurate method of evaluating a patient’s diaphragmatic function at the bedside. Two sonographic techniques have been used for the evaluation of diaphragmatic function; one is the evaluation of diaphragm motion using M-mode US, and the other is an assessment of the change in the thickness of the diaphragm during respiration. Although HD patients showed similar diaphragm thickness during quiet breathing and similar RV to normal controls, they showed lower diaphragm thickness at TLC and lower TF. Mean diaphragmatic excursion and velocity were comparable during quiet breathing; however, they were significantly lower in the HD patients during deep breathing. Changes in the displacement of the diaphragm during the transition from quiet breathing to deep breathing (△m) in HD patients were lower than in controls. Based on these results, we can conclude that HD patients do have a common diaphragmatic dysfunction and that ultrasound is a practical tool for measuring diaphragmatic dysfunction.

Our clinical ultrasound evaluation has confirmed that diaphragm dysfunction occurs in the uraemic condition; however, its functional consequences have been poorly described. Exertional fatigue and breathlessness are perhaps the most common and debilitating symptoms experienced by ESRD patients, but their aetiology remains controversial. The conventional explanation for orthopnea, exertional dyspnoea and fatigue is heart failure and fluid overload. Traditionally, the neuroendocrine response to left ventricular dysfunction results in sodium and water retention, increased left atrial pressure and maintenance of cardiac output through the Starling mechanism^18^. This increase in left atrial pressure produces a parallel rise in pulmonary venous pressure and predisposes the patient to pulmonary congestion and dyspnoea. Failure of the Starling mechanism to increase cardiac output and oxygen delivery to exercising muscle will result in dyspnoea^20^. Although pulmonary congestion may be a major factor in dyspnoea, it is apparent that dyspnoea is not simply related to pulmonary venous congestion and that there are a number of contributing factors^21^. In the fully adjusted model, people who experience dyspnoea have smaller △m. The reader may wonder why we focus on the relationship between diaphragm dysfunction and breathlessness. First, skeletal muscle dysfunction is one of the most common extrapulmonary manifestations of ESRD, and the prevalence of skeletal muscle dysfunction increases with worsening disease severity^22^. Second, the skeletal muscles play a vital role by providing the mechanical basis for breathing and movement. Peripheral and respiratory muscle dysfunction is a significant contributor to breathlessness and decreased functional capacity^23^. Third, skeletal muscle dysfunction not only contributes to symptoms and functional impairment in CKD but also influences prognosis^24^. Most importantly, unlike dysfunctions of the lungs and heart, skeletal muscle dysfunction is potentially remediable through relatively simple interventions such as exercise training^22^. The same result was obtained after considering the capacity load indicator in the dialysis patient population (Supplemental Table 5). This provides an opportunity to significantly improve the symptoms and functional performance of HD patients with breathlessness, who often have limited pharmacological options for treatment of their heart disease.

Hiccuping is a spasmodic involuntary contraction of the diaphragm that triggers sudden inspiration and an abrupt closure of the glottis with a characteristic sound. It is classified as a hiccup attack, persistent hiccup or rebellious or intractable hiccup according to its duration^25^. Currently, there are no reported studies on the incidence and prevalence of persistent and refractory hiccups in HD patients. Any process that affects the afferent, central or efferent components of the proposed reflex arc can trigger hiccups. If possible, the treatment of persistent hiccups should address the immediate cause of the condition. Our data also showed that the symptoms of hiccup were negatively correlated with △m. In general, patients with diaphragmatic dysfunction are more likely to have a hiccup problem. Therefore, increased diaphragm function may reduce the incidence of hiccup in HD patients.

Fatigue is another experience that is associated with poor outcome in HD patients. It was found that △m was negatively correlated with fatigue; that is, individuals with diaphragmatic dysfunction experienced stronger feelings of fatigue. Multiple linear regression model analysis also revealed that fatigue scores were associated with albumin and glucose. We performed the same analysis in the hemodialysis subgroup, and obtained similar results. Therefore, in addition to improving nutritional status and heart function, improving diaphragm function is also an optional measure for reducing fatigue in HD patients.

Several limitations of the present study should be addressed. The first limitation is that selection bias cannot be excluded due to the small sample size; therefore, our findings may not be generalizable to other HD cohorts. Future large prospective trials are required to confirm the relationship between diaphragmatic dysfunction and exercise tolerance in haemodialysis patients. Another limitation is that the clinical symptoms were measured using a questionnaire. Some illiterate patients or patients with blurred vision required assistance from the staff to complete the questionnaire, and the results of the assessment may be inaccurate. In addition, we did not evaluate diaphragm strength using a more sophisticated technique such as magnetic phrenic nerve stimulation.

## Conclusions

The prevalence of diaphragmatic dysfunction was high in HD patients, and it was associated with clinical symptoms, such as fatigue, dyspnoea and hiccup. Ultrasound is a promising tool for monitoring diaphragmatic function and for timely diagnosis. Interventional studies are needed to examine whether targeting diaphragmatic dysfunction can improve clinical symptoms such as dyspnoea, fatigue, and hiccup.

## Materials and Methods

We conducted a cross-sectional study at the Institute of Nephrology, Southeast University.

### Patients

**Figure 1:**
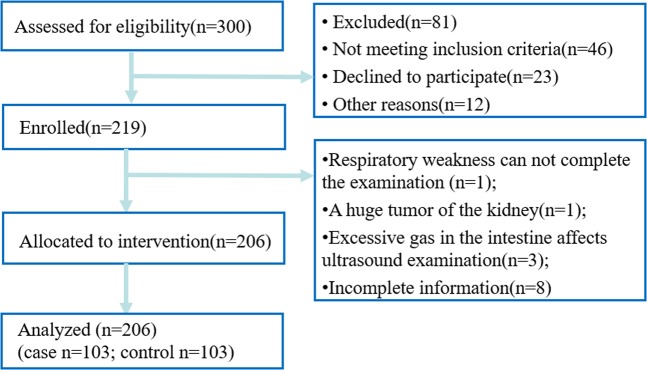
Flow chart of the selection of participants included.

From May 18, 2018 to November 2, 2018, we recruited adults (age > 18 years) who underwent maintenance haemodialysis at the Institute of Nephrology, Southeast University. In accordance with the ratio of 1:1 matching with a normal renal function control group, the exclusion criteria were: (1) acute renal insufficiency; (2) malignant tumours; (3) chronic respiratory disease, including bronchial asthma, COPD, *et al*.; (4) myasthenia gravis or structural damage to the diaphragm (including trauma, surgery, and fistula); and (5) Stroke, dementia, depression or psychosis; (6) Anti-depressive or anti-psychotic drugs; (7) incomplete data. We obtained demographic and medical information on the participants, including gender, age, BMI, smoking, and drinking, comorbidities (hypertension, diabetes mellitus, CHD, CHF), laboratory test results (haemoglobin, serum calcium, phosphorus, potassium, albumin, triglycerides (TG), high-sensitivity C-reactive protein, and serum glucose) and drugs used (Calcium channel blockers (CCB), β blockers, Angiotensin II receptor antagonist (ARB), Angiotensin-converting enzyme inhibitor (ACEI), statins, and glucocorticoids) that may influence muscle metabolism (Fig. 1 shows the flow chart of the selection of participants included). In addition, we recorded the patients’ clinical symptoms, including dyspnoea, hiccup, and fatigue. The Medical Research Council (MRC) dyspnoea scale was used to evaluate the breathlessness of participants^26^. We defined grade 0–2 as no dyspnea, and grade 3–5 as dyspnea. Hiccups were also assessed using a point system. Patients who were completely asymptomatic were recorded as 1, those with acute attacks lasting less than 48 h were recorded as 2, ‘persistent hiccups’ lasting more than 2 days were recorded as 3, and if the attack lasted more than 1 month, it was recorded as 4. When a patient’s score was less than or equal to 2, he or she was recorded as having no hiccuping, and patients with scores greater than 2 were recorded as having hiccups. The assessment of the degree of fatigue relied primarily on the multidimensional fatigue inventory (MFI-20)^27^. Higher scores on the fatigue scale indicated stronger feelings of fatigue.

### Diaphragmatic ultrasound

**Figure 2:**
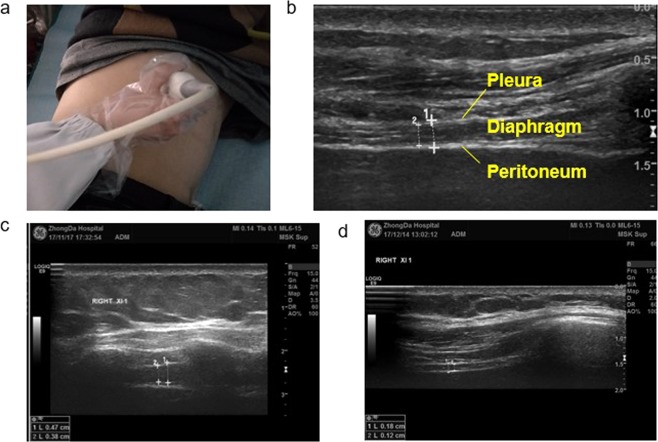
Diaphragm thickness reduction in dialysis patients during deep breathing under ultrasound. (**a**) The probe position for B and M mode diaphragmatic thickness measurements in the zone of apposition using a 6–13 MHz probe. (**b**) B-mode sonography of the diaphragm in the zone of apposition. The diaphragm is visualized as a three-layered structure comprising two parallel echogenic layers of diaphragmatic pleura and peritoneal membranes sandwiching a non-echogenic layer of the diaphragm muscle. (**c**) End-inspiration thickness of the diaphragm at forced vital capacity in a normal person. (**d**) End-inspiration thickness of the diaphragm at forced vital capacity in a dialysis patient.

Diaphragmatic ultrasound was used to evaluate the structure and function of the diaphragm on the right side, which is most commonly used in such evaluations^28^. During the ultrasound examination, the participant assumed a lateral position and breathed independently. Diaphragm thickness was measured using B-mode ultrasound with a linear transducer (6–13 MHz) placed over the diaphragm apposition zone (Fig. 2a) close to the costal phrenic angle between the right anterior and medial axillary lines. The diaphragm thickness was measured from the most superficial hyperechoic line (pleural line) to the deepest hyperechoic line (peritoneal line) (Fig. 2b). We measured the thickness of the diaphragm at FRC, tidal volume (VT), RV, TLC. Three breathing cycles were measured and averaged. We also calculated TF using the following equation: TF = ((Thickness at FRC − Thickness at RV) ∕ Thickness at RV) × 100^24^. TF has been related to lung volume and may be a useful index for evaluating diaphragmatic function^29^.

**Figure 3:**
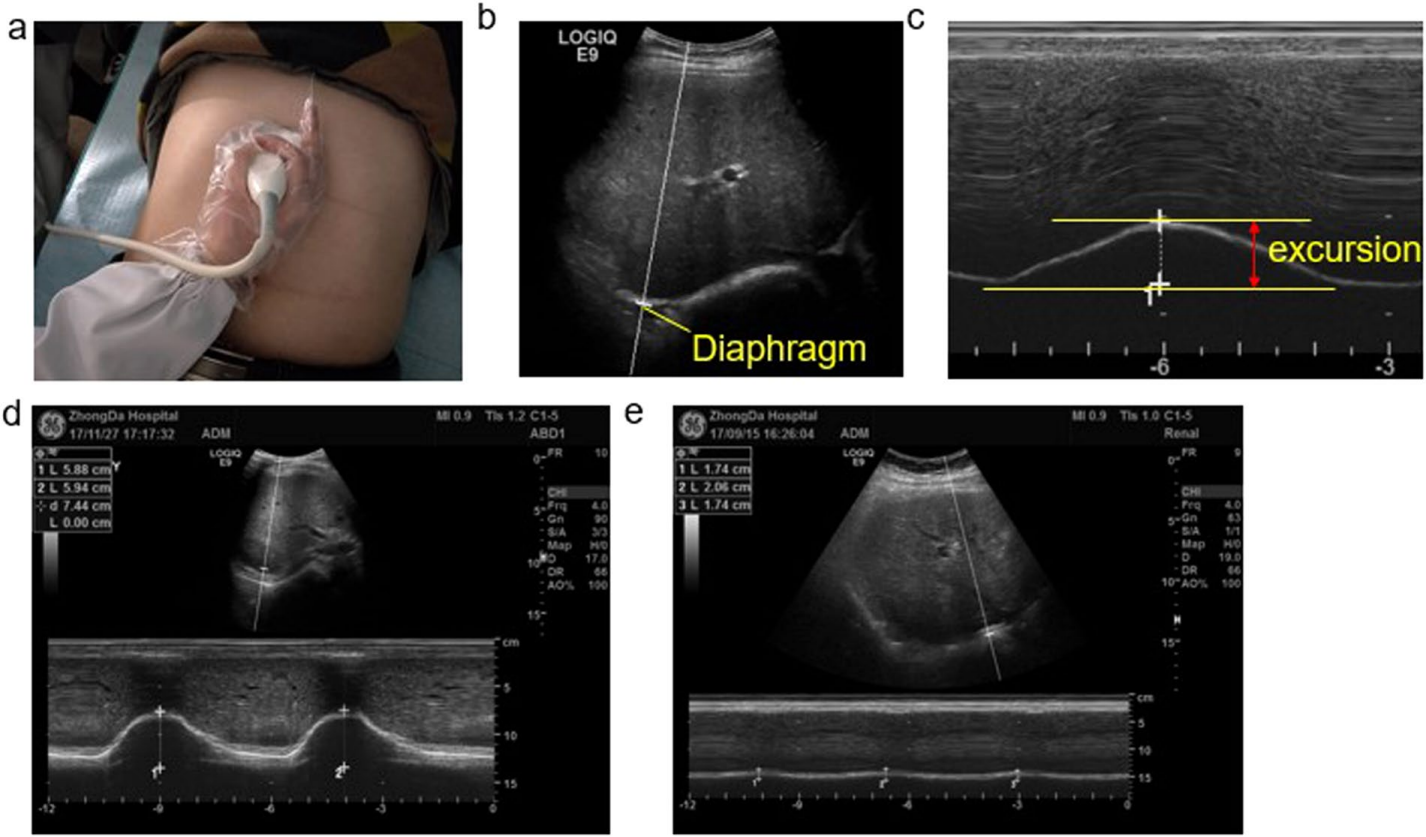
Diaphragm excursion measured by ultrasound decreases in HD patients during deep breathing. (**a**) The appropriate probe position for B and M mode diaphragmatic mobility measurements using a 1–5 MHz probe. (**b**) B-mode diaphragm sonography; the bright line reflects the diaphragm using the anterior subcostal approach. (**c**) M-mode tracing showing the amplitude of excursion during deep breathing. The arrows indicate the beginning and the end of diaphragmatic contraction, and the distance between the arrows indicates the diaphragm excursion. (**d**) Diaphragm excursion in a normal person during deep breathing. (**e**) Diaphragm excursion in a haemodialysis dialysis patient during deep breathing.

The participants assumed a supine position and breathed spontaneously. The ultrasound transducer was placed on the lower edge of the rib arch of the right midclavicular line, and diaphragm excursion was measured at this position (Fig. 3a,b). Then, in the ultrasound M mode, we measured the amplitude of the craniocaudal diaphragm excursion during quiet breathing and deep breathing. The diaphragmatic excursion was measured on the vertical axis of the tracing as the distance from the baseline to the point of maximum height of inspiration on the graph (Fig. 3b). Moreover, we calculated the difference in diaphragmatic displacement between calm breathing and deep breathing, marked as △m. Previous studies have shown that △m can be used as an indicator of diaphragm function^30^. The time of diaphragmatic contraction was defined as the difference between the beginning of inspiration and when the peak was reached during a quiet breath. The diaphragmatic velocity of contraction (cm/s) was calculated as the diaphragmatic movement (cm) divided by the duration of diaphragmatic contraction (s). We recorded the average of three consecutive measurements. Ying-yan Wang performed the US.

### Statistical analyses

Measurement data with normal distribution are expressed as $\bar{{\rm{x}}}$ ± s. Data that did not meet the normal distribution are expressed as medians (P25, P75). For comparisons, continuous variables between groups, the t-test or the Mann-Whitney U test was performed. Fisher’s exact test was used for categorical variables. To determine the association between diaphragm parameters and clinical factors, a multivariable linear regression model was used. A binary logistic regression model was performed to investigate the associations between diaphragm parameters and clinical symptoms. P < 0.05 was considered to be statistically significant. All of the analyses were performed using R (x64, version 3.3.3, R Foundation for Statistical Computing, Vienna, Austria).

### Ethics

The study protocol was approved by the Ethics Committee of Zhongda Hospital affiliated to Southeast University (2018ZDKYSB167), and the study was conducted in accordance with the Helsinki Declaration and Chinese law. The details of the study were explained to each patient; if he or she agreed to participate, a written informed consent was signed.

### Clinical trial registration

Chinese Clinical Trials Registry, http://www.chictr.org.cn, 2018ZDKYSB167. Retrospectively registered on May 18, 2018.

## Supplementary information

Supplementary table

## Data Availability

The datasets generated during and/or analysed during the current study are available from the first author on reasonable request.
