# Supplementary material for: Diaphragmatic dysfunction associates with dyspnoea, fatigue, and hiccup in haemodialysis patients: a cross-sectional study
Source: Sci Rep. 2019 Dec 18;9:19382. doi: 10.1038/s41598-019-56035-4 (PMC6920450; doi:10.1038/s41598-019-56035-4)
Supplement: Supplementary file 1 — Supplementary table [file 41598_2019_56035_MOESM1_ESM.docx]

**Diaphragmatic dysfunction associates with dyspnoea, fatigue, and** **hiccup in haemodialysis patients:** **a cross-sectional study**

Bin Wang, MD^1#^, Email: [wangbinhewei@126.com](mailto:wangbinhewei@126.com); Qing Yin, PhD^1#^, Email: yinqingseu@163.com; Ying-yan Wang, PhD^2#^, Email:[16131443@qq.com](mailto:16131443@qq.com); Yan Tu, PhD^1^, Email: [tuyan_luck@hotmail.com](mailto:tuyan_luck@hotmail.com); Yuchen Han,MD^1^, Email:[njhanyuchen@qq.com](mailto:njhanyuchen@qq.com); Min Gao, MD^1^,[gaomin1220@yeah.net](mailto:gaomin1220@yeah.net); Mingming Pan, MD^1^, [mingmingpan2001@163.com](mailto:mingmingpan2001@163.com); Yan Yang, MD^1^, Email: [yangyan_home@163.com](mailto:yangyan_home@163.com); Yufang Xue, PhD^1^, Email: [376980635@qq.com](mailto:376980635@qq.com); Li Zhang^1^, [jiangshengzhe2008@163.com](mailto:jiangshengzhe2008@163.com); Liuping Zhang^1^, [shery9926@sina.com](mailto:shery9926@sina.com); Hong Liu, PhD^1^, Email: [jstzliu@sina.com](mailto:jstzliu@sina.com); Rining Tang, MD^1^, [tangrn77@163.com](mailto:tangrn77@163.com); Xiaoliang Zhang, M.D^1^., Email: [tonyxlz@163.com](mailto:tonyxlz@163.com); Jingjie xiao_，_MD^3^, [jingjie1@ualberta.ca](mailto:jingjie1@ualberta.ca); Xiaonan H. Wang, M.D^4^, Email: [xwang03@emory.edu](mailto:xwang03@emory.edu); Bi-Cheng Liu, MD, PhD^1^, [liubc64@163.com](mailto:liubc64@163.com).

# These authors contributed equally to this work.

* Corresponding author: Professor Bi-Cheng Liu, MD, PhD ;

Email: [liubc64@163.com](mailto:liubc64@163.com);

Institute of Nephrology, Zhongda Hospital, Southeast University School of Medicine No. 87, Dingjiaqiao Road, Gulou District, Nanjing, Jiangsu Province, China.

Supplemental Table 1 Multiple linear regression assignment table

| Variable | Assignment |
| --- | --- |
| Sex | Male = 1; female = 2 |
| Age | Continuous variable |
| Height | Continuous variable |
| Weight | Continuous variable |
| BMI | Continuous variable |
| Smoking | Yes = 1; No = 0 |
| Hb | Continuous variable |
| Alb | Continuous variable |
| TG | Continuous variable |
| Glu | Continuous variable |
| CCB | Yes = 1; No = 0 |
| β blockers | Yes = 1; No = 0 |
| Hypertension | Yes = 1; No = 0 |
| DM | Yes = 1; No = 0 |
| CHD | Yes = 1; No = 0 |
| CHF | Yes = 1; No = 0 |
| Glucocorticoids | Yes = 1; No = 0 |
| TdiTLC | Continuous variable |
| DMTLC | Continuous variable |
| TF | Continuous variable |
| △m | Continuous variable |
| XD weight | Continuous variable |

Hb, Haemoglobin; Alb, Albumin; TG, Triglycerides; Glu, Glucose; CCB, Calcium channel blockers; β blocker, Beta blockers; DM, Diabetes mellitus; CHD, Coronary heart disease; CHF, Chronic heart failure; TdiTLC, End-inspiration thickness of the diaphragm at total lung capacity (TLC); DMTLC, Diaphragm excursion at TLC ; TF, Thickening fraction; △m = DMFRC-DMTLC; XD.weight: interdialysis weight gain rate.

Supplemental Table 2 Multiple linear regression of diaphragm parameters and other factors in the HD subgroup

|  | DMTLC | | TF | | TdiTLC | | △m | | velocity2 | |
| --- | --- | --- | --- | --- | --- | --- | --- | --- | --- | --- |
|  | Coefficient | *P* | Coefficient | *P* | Coefficient | *P* | Coefficient | *P* | Coefficient | *P* |
| Age | -.0296072 | 0.101 | -.0017133 | 0.754 | -.0008771 | 0.479 | -.036794 | *0.007* | -.0137191 | 0.365 |
| Sex | -.2017707 | 0.802 | .185905 | 0.450 | -.1750068 | ***0.002*** | .5137178 | **0.395** | -.5538769 | 0.415 |
| Height | -.0506733 | 0.287 | .0029449 | 0.839 | -.0033718 | 0.305 | -.0215244 | 0.545 | -.0791119 | 0.051 |
| Weight | .0370444 | 0.081 | .0041237 | 0.521 | .0027325 | **0.062** | .0242034 | 0.126 | .0316611 | 0.076 |
| Smoking | -.2283791 | 0.713 | -.168764 | 0.374 | .0620929 | 0.150 | -.5796546 | 0.214 | .2337518 | 0.655 |
| Hb | -.0065117 | **0.599** | -.0065717 | 0.085 | -.0013434 | 0.119 | -.0051835 | **0.576** | -.0087774 | **0.401** |
| Alb | -.0138896 | 0.820 | -.0069266 | 0.710 | .0006657 | 0.874 | .0145591 | 0.750 | -.0402454 | 0.434 |
| TG | .0660052 | 0.764 | -.0002163 | 0.997 | .013387 | 0.378 | .0543525 | 0.741 | -.0973007 | 0.600 |
| CHD | .1090175 | 0.886 | -.4958285 | ***0.035*** | -.0043921 | 0.933 | -.5245006 | 0.357 | .3769623 | 0.556 |
| CHF | -.4730541 | 0.481 | .0038007 | 0.985 | -.0088181 | 0.849 | .0967148 | 0.847 | -.2005888 | 0.723 |
| Glucocorticoids | 1.954052 | 0.200 | -.0112776 | 0.981 | -.0967385 | 0.356 | 2.973496 | 0.010 | .7570502 | 0.554 |

Hb, Haemoglobin; Alb, Albumin; TG, Triglycerides; CHD, Coronary heart disease; CHF, Chronic heart failure.

Supplemental Table 3 Clinical findings

| Variable | All patients  (*n* = 206) | Maintenance HD  (*n* = 103) | Controls  (*n* = 103) | P value |
| --- | --- | --- | --- | --- |
| Dyspnoea | 27/206 | 27/103 | 0/103 | *P*<*0.001* |
| Hiccup | 17/206 | 16/103 | 1/103 | *P*<*0.001* |
| Fatigue (Score) | 45.39±17.94 | 58.65±14.64 | 32.14±8.17 | *P*<*0.001* |

Supplemental Table 4 Logistic regression assignment table

| Variable | Assignment |
| --- | --- |
| Sex | Male = 1; female = 2 |
| Age | Continuous variable |
| BMI | Continuous variable |
| Smoking | Yes = 1; No = 0 |
| Hb | Continuous variable |
| Alb | Continuous variable |
| TG | Continuous variable |
| Glu | Continuous variable |
| CCB | Yes = 1; No = 0 |
| β blockers | Yes = 1; No = 0 |
| Hypertension | Yes = 1; No = 0 |
| DM | Yes = 1; No = 0 |
| CHD | Yes = 1; No = 0 |
| CHF | Yes = 1; No = 0 |
| Glucocorticoids | Yes = 1; No = 0 |
| TdiTLC | Continuous variable |
| DMTLC | Continuous variable |
| TF | Continuous variable |
| △m | Continuous variable |
| XD weight | Continuous variable |

BMI, Body Mass Index; Hb, Haemoglobin; Alb, Albumin; TG, Triglycerides; Glu, Glucose; CCB, Calcium channel blockers; β blocker, Beta blockers; DM, Diabetes mellitus; CHD, Coronary heart disease; CHF, Chronic heart failure; TdiTLC, End-inspiration thickness of the diaphragm at total lung capacity (TLC); DMTLC, Diaphragm excursion at TLC ; TF, Thickening fraction; △m = DMFRC-DMTLC; XD.weight: interdialysis weight gain rate.

Supplemental Table 5 A binary logistic regression model of dyspnoea and other factors (HD)

| Dyspnoea | OR | [95% Conf. | Interval] | P>\|z\| |
| --- | --- | --- | --- | --- |
| Age | 1.03096 | 0.966773 | 1.099409 | 0.353 |
| TdiTLC | 25.36004 | 0.211323 | 3043.359 | 0.186 |
| DMTLC | 1.52791 | 0.785999 | 2.970114 | 0.211 |
| △m | 0.377462 | 0.142129 | 1.00245 | *0.051* |
| TF | 1.922047 | 0.558971 | 6.609046 | 0.3 |
| Hypertension | 8.388531 | 0.037553 | 1873.816 | 0.441 |
| Hb | 1.039144 | 0.994871 | 1.085387 | 0.084 |
| Alb | 0.800738 | 0.650221 | 0.986099 | *0.036* |
| Glu | 0.784872 | 0.561198 | 1.097695 | 0.157 |
| CCB | 2.078407 | 0.363937 | 11.86958 | 0.411 |
| CHD | 2.434313 | 0.270245 | 21.92779 | 0.428 |
| CHF | 93.86871 | 4.470309 | 1971.079 | *0.003* |
| DM | 0.277954 | 0.032985 | 2.342254 | 0.239 |
| XDweight | 1.117438 | 0.960347 | 1.300225 | 0.151 |

TdiTLC, End-inspiration thickness of the diaphragm at total lung capacity (TLC); DMTLC, Diaphragm excursion at TLC ;△m = DMFRC-DMTLC; TF, Thickening fraction; Hb, Haemoglobin; Alb, Albumin; Glu, Glucose; CCB, Calcium channel blockers; CHD, Coronary heart disease; CHF, Chronic heart failure; DM, Diabetes mellitus; XDweight: interdialysis weight gain rate.

Supplemental Table 6 Multiple linear regression of fatigue and other factors (HD)

| Fatigue | Coefficient | [95% Conf. | Interval] | P>t |
| --- | --- | --- | --- | --- |
| △m | -2.40931 | -4.33177 | -0.48685 | ***0.015*** |
| TF | 0.06228 | -5.59742 | 5.721983 | 0.983 |
| TdiTLC | 6.981013 | -15.7298 | 29.69184 | 0.542 |
| Hypertension | -0.60088 | -13.1711 | 11.9693 | 0.924 |
| BMI | 0.361094 | -0.62495 | 1.347134 | 0.468 |
| Hb | 0.163157 | -0.00163 | 0.327947 | *0.052* |
| Alb | -1.20728 | -1.93433 | -0.48023 | ***0.001*** |
| Glu | 0.591652 | -0.53646 | 1.719767 | 0.3 |
| CCB | 2.37244 | -4.26749 | 9.012369 | 0.479 |
| ARB | 1.777496 | -5.41532 | 8.970313 | 0.624 |
| β blockers | -1.13189 | -7.71788 | 5.454104 | 0.733 |
| XDweight | 0.502459 | -0.09829 | 1.103211 | 0.1 |

△m = DMFRC-DMTLC; TF, Thickening fraction; TdiTLC, End-inspiration thickness of the diaphragm at total lung capacity (TLC); BMI, Body Mass Index; Hb, Haemoglobin; Alb, Albumin; Glu, Glucose; CCB, Calcium channel blockers; ARB, Angiotensin II receptor antagonist; β blocker, Beta blockers; XD.weight: interdialysis weight gain rate.

Supplemental Table 7 A binary logistic regression model of hiccups and other factors (HD).

| Hiccups | OR | [95% Conf. | Interval] | P>\|z\| |
| --- | --- | --- | --- | --- |
| DMTLC(cm) | 1.673443 | 0.801051 | 3.495917 | 0.171 |
| △m(cm) | 0.205341 | 0.062102 | 0.678965 | ***0.009*** |
| TdiRV(cm) | 2.83257 | 8.72e-07 | 9202248 | 0.892 |
| TdiTLC(cm) | 8.330706 | 0.007295 | 9514.042 | 0.555 |
| Hypertension | 0.640038 | 0.084961 | 22.53109 | 0.924 |
| Hb(g/L) | 0.979373 | 0.937415 | 1.02321 | 0.351 |
| Alb(g/L) | 0.957301 | 0.784528 | 1.168123 | 0.667 |
| Glu (mmol/L) | 0.975790 | 0.751890 | 1.266363 | 0.854 |
| CCB | 2.121985 | 0.312946 | 14.3885 | 0.441 |
| ARB | 3.680397 | 0.556426 | 24.34346 | 0.176 |
| β blockers | 0.306391 | 0.038469 | 2.440321 | 0.264 |
| CHD | 3.727363 | 0.234774 | 59.17719 | 0.351 |
| CHF | 0.385935 | 0.017885 | 8.32807 | 0.544 |
| DM | 2.623678 | 0.435711 | 15.79876 | 0.292 |
| XDweight | 1.16011 | .9588608 | 1.403599 | 0.127 |

DMTLC, Diaphragm excursion at TLC ;△m = DMFRC-DMTLC; TdiRV, End-expiration thickness of the diaphragm at residual capacity (RV); TdiTLC, End-inspiration thickness of the diaphragm at total lung capacity (TLC); Hb, Haemoglobin; Alb, Albumin; Glu, Glucose; CCB, Calcium channel blockers; ARB, Angiotensin II receptor antagonist; β blocker, Beta blockers; CHD, Coronary heart disease; CHF, Chronic heart failure; DM, Diabetes mellitus; XD.weight: interdialysis weight gain rate.
